# Evaluation of post-acute care and one-year outcomes among Medicare beneficiaries with hip fractures: a retrospective cohort study

**DOI:** 10.1186/s12916-023-02958-9

**Published:** 2023-07-03

**Authors:** Melissa R. Riester, Francesca L. Beaudoin, Richa Joshi, Kaleen N. Hayes, Meghan A. Cupp, Sarah D. Berry, Andrew R. Zullo

**Affiliations:** 1 Department of Health Services, Policy, and Practice, Brown University School of Public Health, 200 Dyer Street, Box 2013, Providence, RI 02912 USA; 2 Department of Epidemiology, Brown University School of Public Health, Providence, RI USA; 3 Graduate Department of Pharmaceutical Sciences, Faculty of Pharmacy, University of Toronto Leslie Dan, Toronto, ON Canada; 4 Hinda and Arthur Marcus Institute for Aging Research, Hebrew SeniorLife, Roslindale, MA USA; 5 Department of Medicine, Beth Israel Deaconess Medical Center, Boston, MA USA; 6 Harvard Medical School, Boston, MA USA; 7 Center of Innovation in Long-Term Services and Supports, Providence Veterans Affairs Medical Center, Providence, RI USA

**Keywords:** Hip fractures, Patient discharge, Rehabilitation, Skilled nursing facilities, Subacute care

## Abstract

**Background:**

Post-acute care (PAC) services after hospitalization for hip fracture are typically provided in skilled nursing facilities (SNFs), inpatient rehabilitation facilities (IRFs), or at home via home health care (HHC). Little is known about the clinical course following PAC for hip fracture. We examined the nationwide burden of adverse outcomes by PAC setting in the year following discharge from PAC for hip fracture.

**Methods:**

This retrospective cohort included Medicare Fee-for-Service beneficiaries > 65 years who received PAC services in U.S. SNFs, IRFs, or HHC following hip fracture hospitalization between 2012 and 2018. Individuals who had a fall-related injury (FRI) during PAC or received PAC services in multiple settings were excluded. Primary outcomes included FRIs, all-cause hospital readmissions, and death in the year following discharge from PAC. Cumulative incidences and incidence rates for adverse outcomes were reported by PAC setting. Exploratory analyses examined risk ratios and hazard ratios between settings before and after inverse-probability-of-treatment-weighting, which accounted for 43 covariates.

**Results:**

Among 624,631 participants (SNF, 67.78%; IRF, 16.08%; HHC, 16.15%), the mean (standard deviation) age was 82.70 (8.26) years, 74.96% were female, and 91.30% were non-Hispanic White. Crude incidence rates (95%CLs) per 1000 person-years were highest among individuals receiving SNF care for FRIs (SNF, 123 [121, 123]; IRF, 105 [102, 107]; HHC, 89 [87, 91]), hospital readmission (SNF, 623 [619, 626]; IRF, 538 [532, 544]; HHC, 418 [414, 423]), and death (SNF, 167 [165, 169]; IRF, 47 [46, 49]; HHC, 55 [53, 56]). Overall, rates of adverse outcomes generally remained higher among SNF care recipients after covariate adjustment. However, inferences about the group with greater adverse outcomes differed for FRIs and hospital readmissions based on risk ratio or hazard ratio estimates.

**Conclusions:**

In this retrospective cohort study of individuals hospitalized for hip fracture, rates of adverse outcomes in the year following PAC were common, especially among SNF care recipients. Understanding risks and rates of adverse events can inform future efforts to improve outcomes for older adults receiving PAC for hip fracture. Future work should consider calculating risk and rate measures to assess the influence of differential time under observation across PAC groups.

**Supplementary Information:**

The online version contains supplementary material available at 10.1186/s12916-023-02958-9.

## Background

Hip fractures are a major source of disability for older adults. Over 300,000 older adults in the United States are hospitalized for hip fractures each year [1]. More than 90% may require post-acute care (PAC) in skilled nursing facilities (SNFs), inpatient rehabilitation facilities (IRFs), or at home with home health care (HHC) after hospital discharge to facilitate medical recovery, restore physical function, and maximize independence [2–6]. Despite the beneficial effects of PAC, individuals with hip fracture often experience adverse outcomes after discharge from PAC, such as loss of functional independence, hospital readmission, and death [7–13].

PAC settings differ in their structure and services provided [14]. In general, the selection of PAC setting following hip fracture is determined by the need for rehabilitation services, functional status, management of medical conditions, social support, proximity to patient’s home, insurance coverage, and personal preference [2]. For example, IRFs are required to provide intensive rehabilitation services, with at least three hours of therapy daily for at least 5 days per week, whereas SNFs and HHC do not have these minimum therapy hour requirements. Characteristics of patients receiving care in each setting also differ [4, 15–18]. Individuals with a hip fracture who receive care in SNFs generally have the greatest medical complexity, cognitive impairment, and physical impairment. These patients’ families and other caregivers often do not feel comfortable with or capable of providing 24-h care at home.

Prior studies examining adverse outcomes following hip fracture have focused on the post-hospitalization period, starting follow-up either at the time of hospital discharge or entry into PAC [4, 10, 13, 16, 19–29]. Since goals of PAC for hip fracture include improving physical function, facilitating medical recovery, and successfully discharging patients to the community, understanding the clinical course for patients with a hip fracture following PAC discharge would provide valuable information to patients, clinicians, researchers, and policymakers. Accurate nationwide estimates of adverse outcomes following discharge from hip fracture PAC across PAC settings could inform future research efforts, quality improvement interventions, and policies aimed at improving outcomes.

Our primary objective was to estimate risks and rates of fall-related injuries (FRIs), hospital readmission, and death in the year following PAC discharge using a nationwide sample of Medicare Fee-for-Service beneficiaries discharged to SNFs, IRFs, or HHC following a hip fracture hospitalization. We hypothesized that individuals receiving SNF care would have the highest rates of adverse outcomes.

## Methods

### Data sources

We linked Medicare claims to the Centers for Medicare and Medicaid Services’ publicly available Provider of Service File. The Medicare Beneficiary Summary File (MBSF) included demographic and enrollment information [30]. The timing and location of health services utilization was determined using a validated Residential History File that employs Medicare, Outcome and Assessment Information Set (OASIS), Minimum Data Set (MDS), and other data [31]. Medicare Provider Analysis and Review (MedPAR) claims supplied information on inpatient admissions and diagnoses [32]. Medicare Part D claims contained data on prescription drug dispensings. The Centers for Medicare & Medicaid Services’ Provider of Service File for Hospital and Non-hospital Facilities included information on facilities where participants were hospitalized for hip fracture [33]. The Brown University Institutional Review Board approved the study. Informed consent was not required.

### Study design and population

This nationally representative retrospective longitudinal cohort study included over one million Medicare Fee-for-Service beneficiaries hospitalized with a hip fracture between 2012 and 2018. Eligible participants were > 65 years old, hospitalized for hip fracture (based on the principal diagnosis position on the inpatient claim), continuously enrolled in Medicare Parts A, B, and D during PAC and for 12 months prior to the hip fracture hospitalization, discharged to a PAC setting of interest following hospitalization, and discharged alive from PAC. Individuals who left against medical advice during their hip fracture hospitalization, had an FRI during PAC, and those who did not reside within the 48 contiguous states were excluded.

### PAC setting

We identified participants who were discharged from the hip fracture hospitalization to a SNF, IRF, or HHC for PAC. Individuals who received PAC in long-term acute care hospitals or multiple institutional settings (i.e., IRF and SNF) were excluded due to limited sample size (0.07% and 2.4% of hip fracture hospitalizations in our sample, respectively) and concerns that there would be limited statistical power to detect a difference in outcomes for comparisons to these groups. We excluded participants who were discharged from the hip fracture hospitalization to critical access hospitals, hospice, and nursing homes (long-term care only) since these settings are generally not intended to deliver PAC.

### Outcomes

The primary outcomes were FRIs, all-cause hospital readmission, and death in the year following discharge from PAC. We defined FRIs using a previously published algorithm (Additional file 1: Table S1) [34]. This algorithm was based on studies that identified fractures and FRIs using inpatient claims data (sensitivity 62.1–100%, positive predictive value 53.2–98.6% depending on the study and site of injury) [35–37]. Secondary analyses examined specific FRIs, including hip fracture, lower extremity fracture, axial fracture, upper extremity fracture, and intracranial bleeding.

### Follow-up

Start of follow-up (time zero) began the day of discharge from SNFs and IRFs or 60 days after hospital discharge for participants receiving HHC. We could not reliably ascertain the exact date that HHC services ended because the dates of discharge from HHC were not consistently reported on OASIS assessments. Thus, we chose to begin follow-up 60 days after hospital discharge as this aligned with Medicare payment policy for an episode of HHC during the study period [38]. Participants were followed from time zero until each outcome of interest, if it occurred. If the outcome of interest did not occur, we recorded the time until disenrollment from Medicare Parts A, B, or D, death (for FRI and hospital readmission analyses), 12 months from PAC discharge, or end of the study period (December 31, 2019), whichever occurred first.

### Participant and hospital characteristics

Demographics (e.g., age, sex, and race/ethnicity) and dual Medicare/Medicaid enrollment status were obtained from the MBSF. Participant diagnoses were ascertained from the MedPAR hip fracture hospitalization record and conditions were categorized based on the Healthcare Cost and Utilization Project Clinical Classifications Software [39]. MedPAR was also used to obtain characteristics from the index hip fracture hospitalization, including length of stay, intensive care unit use, complications during hospitalization (urinary tract infection, pressure ulcer, pneumonia), fracture management (e.g., partial joint replacement, internal fixation), the Claims-based Frailty Index, and Gagne Combined Comorbidity Score [40, 41]. Prior medication use was defined as at least one dispensing during the 12 months prior to the hip fracture hospitalization in Part D. We ascertained opioids, non-steroidal anti-inflammatory drugs, gabapentinoids, and benzodiazepines as these medication classes have either been associated with falls and fracture or indicate the presence of pain, which itself is a risk factor for falls [42–45]. Characteristics of hospitals where participants were treated for hip fracture included number of beds, medical school affiliation, urban/rural location, region, and ownership. All characteristics were measured on or before the date of hospital discharge.

### Statistical analyses

Our primary objective was to provide nationwide estimates of the burden of adverse outcomes in the year following discharge from hip fracture PAC. We calculated crude one-year cumulative incidences (i.e., risks) and incidence rates (IRs) for FRIs, all-cause hospital readmission, death, and specific FRIs separately according to PAC setting. IRs were calculated per 1,000 person-years (PYs) of follow-up time. We calculated 95% confidence limits (CLs) for IRs using the non-parametric bootstrap (*n* = 1,000 resamplings) and the percentile method.

We also conducted exploratory analyses to compare risks and rates of adverse outcomes between PAC settings, before and after adjustment for patient- and hospital-level characteristics. Adjusted risk ratio (RR) and hazard ratio (HR) estimates were calculated using stabilized inverse-probability-of-treatment-weighted (IPW) modified Poisson and Cox regression models with bootstrap 95%CLs [46]. Crude and adjusted risk differences and rate differences were also estimated. The probabilities (i.e., propensity scores) used to construct the IPWs were estimated via a multinomial logistic regression model, where receipt of PAC in SNF, IRF, or HHC were outcomes [47]. We accounted for 43 covariates (Additional file 1: Table S2) and the multi-level C-statistic of the model was 0.66 [48, 49]. The IPWs were properly distributed (mean [SD], 1.00 (0.43); minimum–maximum, 0.27–10.80). Covariate balance was assessed using standardized mean differences (SMDs). We conducted a separate analysis using cause-specific hazard regression models to account for the competing risk of death for FRI and hospital readmission outcomes.

### Sensitivity analysis

A quantitative bias analysis was conducted by estimating E-values for crude and adjusted HRs and RRs [50, 51].

### Software

Data were analyzed using SAS version 9.4 (SAS Institute, Inc., Cary, NC) and Stata version 17 (StataCorp LLC, College Station, TX).

## Results

### Study population

Among 624,631 Medicare beneficiaries who experienced a hip fracture hospitalization between January 1, 2012 and December 31, 2018 and were discharged to SNFs, IRFs, or HHC for PAC, the mean (standard deviation [SD]) age was 82.70 (8.26) years, 468,247 (74.96%) participants were female, and 570,284 (91.30%) were non-Hispanic White (Table 1). There were 423,347 (67.78%) recipients of SNF care, 100,411 (16.08%) recipients of IRF care, and 100,873 (16.15%) recipients of HHC (Additional file 1: Figure S1). The median (Quartile 1, Quartile 3) length of PAC stay was 37 (20, 63) days for SNF care and 13 (9, 16) days for IRF care.

**Table 1:** Characteristics of individuals receiving post-acute after hip fracture hospitalization, 2012–2018

**Characteristics**	**Overall** (*N* = 624,631)	**SNF** (*n* = 423,347)	**IRF** (*n* = 100,411)	**HHC** (*n* = 100,873)
**Demographics**				
Age, mean (SD), years	82.70 (8.26)	83.76 (8.15)	80.59 (7.95)	80.33 (8.13)
Female Sex	468,247 (74.96)	322,011 (76.06)	72,672 (72.37)	73,564 (72.93)
Race/ethnicity				
Non-Hispanic White	570,284 (91.30)	388,748 (91.83)	91,014 (90.64)	90,522 (89.74)
Non-Hispanic Black	24,482 (3.92)	16,048 (3.79)	3,897 (3.88)	4,537 (4.50)
Hispanic	11,032 (1.77)	6,450 (1.52)	2,325 (2.32)	2,257 (2.24%)
Other^a^	17,004 (2.72)	11,038 (2.61)	2,825 (2.81)	3,141 (3.11)
Unknown	1,829 (0.29)	1,063 (0.25)	350 (0.35)	416 (0.41)
Dual Medicare/Medicaid enrollment	161,103 (25.79)	123,662 (29.21)	18,724 (18.65)	18,717 (18.56)
**Calendar year of fracture**				
2012	81,465 (13.06)	54,452 (12.86)	14,703 (14.64)	12,310 (12.20)
2013	85,178 (13.66)	57,482 (13.58)	14,425 (14.37)	13,271 (13.16)
2014	94,679 (15.16)	63,700 (15.05)	16,049 (15.98)	14,930 (14.80)
2015	93,624 (14.99)	63,429 (14.98)	14,862 (14.80)	15,333 (15.20)
2016	88,836 (14.22)	60,397 (14.27)	13,343 (13.29)	15,096 (14.97)
2017	93,329 (14.94)	62,805 (14.84)	13,837 (13.78)	16,687 (16.54)
2018	87,520 (14.01)	61,082 (14.43)	13,192 (13.14)	13,246 (13.13)
**Conditions**^b^				
Acute myocardial infarction	4,924 (0.79)	3,589 (0.85)	745 (0.74)	590 (0.59)
Acute phlebitis, thrombophlebitis, or thromboembolism	2,440 (0.39)	1,730 (0.41)	3,564 (3.55)	356 (0.35)
Anemia	207,434 (33.21)	143,277 (33.84)	34,957 (34.81)	29,200 (28.95)
Asthma	16,211 (2.60)	10,579 (2.50)	3,009 (3.00)	2,623 (2.60)
Cancer	60,901 (9.75)	40,772 (9.63)	10,903 (10.86)	9,226 (9.15)
Cardiac dysrhythmias	96,617 (15.47)	68,536 (16.19)	15,780 (15.72)	12,301 (12.19)
Cerebrovascular disease	22,610 (3.62)	16,052 (3.79)	3,611 (3.60)	2,947 (2.92)
Chronic kidney disease	47,418 (7.59)	33,261 (7.86)	7,088 (7.06)	7,069 (7.01)
Chronic obstructive pulmonary disease and bronchiectasis	58,283 (9.33)	39,628 (9.36)	10,026 (9.98)	8,629 (8.55)
Coronary atherosclerosis and other heart diseases	37,778 (6.05)	26,337 (6.22)	5,939 (5.91)	5,502 (5.45)
Dementia, delirium, and other cognitive disorders	100,408 (16.07)	83,212 (19.66)	7,288 (7.26)	9,908 (9.82)
Diabetes mellitus	157,873 (25.27)	106,761 (25.22)	26,596 (26.49)	24,516 (24.30)
Gout and other crystal arthropathies	10,399 (1.66)	7,036 (1.66)	1,858 (1.85)	1,505 (1.49)
Heart valve disorders	41,128 (6.58)	29,093 (6.87)	6,907 (6.88)	5,128 (5.08)
Hypertension	256,381 (41.05)	173,440 (40.97)	44,140 (43.96)	38,801 (38.47)
Liver disease	7,048 (1.13)	4,741 (1.12)	1,276 (1.27)	1,031 (1.02)
Low back pain	4,433 (0.71)	2,817 (0.67)	721 (0.72)	895 (0.89)
Other musculoskeletal pain	13,758 (2.20)	9,405 (2.22)	2,078 (2.07)	2,275 (2.26)
Obesity	15,294 (2.45)	9,979 (2.36)	2,796 (2.78)	2,519 (2.50)
Opioid-related disorders	7,646 (1.22)	5,504 (1.30)	993 (0.99)	1,149 (1.14)
Osteoarthritis	58,690 (9.40)	39,377 (9.30)	10,310 (10.27)	9,003 (8.93)
Peripheral and visceral atherosclerosis	19,765 (3.16)	13,190 (3.12)	2,952 (2.94)	3,622 (3.59)
Phlebitis, thrombophlebitis, and thromboembolism	15,675 (2.51)	11,083 (2.62)	2,721 (2.71)	1,871 (1.85)
Pulmonary heart disease	21,492 (3.44)	15,464 (3.65)	3,469 (3.45)	2,559 (2.54)
Rheumatoid arthritis and related disease	9,375 (1.50)	6,179 (1.46)	1,621 (1.61)	1,575 (1.56)
Schizophrenia and other psychotic disorders	7,806 (1.25)	6,281 (1.48)	817 (0.81)	708 (0.70)
Thyroid disorder	81,155 (12.99)	56,267 (13.29)	13,489 (13.43)	11,399 (11.30)
Frailty index^c^				
Robust	162,414 (26.00)	90,007 (21.26)	34,219 (34.08)	38,188 (37.86)
Prefrail	411,929 (65.95)	292,117 (69.00)	61,959 (61.71)	57,853 (57.35)
Mildly-to-severely frail	50,288 (8.05)	41,223 (9.74)	4,233 (4.22)	4,832 (4.79)
Gagne comorbidity score, mean (SD)^d^	3.02 (2.32)	3.21 (2.36)	2.66 (2.22)	2.56 (2.12)
**Medication use before the hip fracture hospitalization**^e^				
Opioids	70,034 (11.06)	50,297 (11.88)	9,350 (9.31)	10,387 (9.51)
NSAIDs	20,061 (3.17)	13,637 (3.22)	2,999 (2.99)	3,425 (3.14)
Gabapentinoids	30,674 (4.85)	22,410 (5.29)	3,952 (3.94)	4,312 (3.95)
Benzodiazepines	42,617 (6.73)	31,745 (7.50)	4,903 (4.88)	5,969 (5.47)
**Hip fracture hospitalization characteristics**				
Length of stay, mean, days	5.15 (2.92)	5.27 (2.99)	4.87 (2.60)	4.93 (2.93)
Hospital complications				
Urinary tract infections	114,237 (18.29)	85,262 (20.14)	14,567 (14.51)	14,408 (14.28)
Pressure ulcer of skin	6,090 (0.97)	4,722 (1.12)	658 (0.66)	710 (0.70)
Pneumonia	22,445 (3.59)	16,713 (3.95)	2,921 (2.91)	2,811 (2.79)
Fracture management^f^				
Partial or total joint replacement	203,080 (32.51)	133,697 (31.58)	36,280 (36.13)	33,103 (32.81)
Internal fixation (any) or external fixation (open or percutaneous approach)	333,871 (53.45)	228,016 (53.86)	52,391 (52.18)	53,464 (53.00)
Other surgical management	1,503 (0.24)	983 (0.23)	261 (0.26)	259 (0.27)
Non-surgical management	307,812 (49.28)	214,702 (50.72)	48,281 (48.08)	44,829 (44.44)
ICU use during hip fracture hospitalization	92,067 (14.74)	64,688 (15.28)	15,541 (15.48)	11,838 (11.74)

Reports number (%), unless otherwise stated

*Abbreviations*: *HHC* Home Health Care, *ICU* Intensive Care Unit, *IRF* Inpatient Rehabilitation Facilities, *NSAIDs* Non-Steroidal Anti-Inflammatory Drugs, *SNF* Skilled Nursing Facilities

^a^Participants identified in the Medicare Beneficiary Summary File as North American Native, Asian, or Other race using the Research Triangle Institute (RTI) Race Code were categorized as “Other” race/ethnicity

^b^Represents the conditions documented on the hip fracture hospitalization claim

^c^Measured using the Claims-based Frailty Index and categorized as: < 0.15 (robust), 0.15–0.24 (prefrail), ≥ 0.25 (mildly-to-severely frail)

^d^Measured using the Gagne Combined Comorbidity Score, ranging from -2 to 26, where higher scores indicate greater multimorbidity

^e^Medication use was defined as at least one dispensing in the 12 months prior to the hip fracture hospitalization

^f^Fracture management was ascertained from International Classification of Diseases, Ninth and Tenth Revision procedure codes documented during the hip fracture hospitalization. Participants could be represented in more than one fracture management category

Before IPW, which balanced measured covariates well (Additional file 1: Table S2), individuals receiving PAC in SNFs were more likely to be older (SNF, 83.76 [8.15] years; IRF, 80.59 [7.95]; HHC, 80.33 [8.13]) and have dual Medicare/Medicaid enrollment (SNF, 29.21%; IRF, 18.65%; HHC, 18.56%) (Table 1). A majority of diagnoses were similar in their distributions between PAC settings; however, a greater proportion of individuals receiving PAC in SNFs had a diagnosis of dementia, delirium, or other cognitive disorders (SNF, 19.66%; IRF, 7.26%; HHC, 9.82%). Individuals receiving SNF care were also more likely to be frail (SNF, 9.74%; IRF, 4.22%; HHC, 4.79%) and multimorbid (comorbidity score, mean [SD]: SNF, 3.21 [2.36]; IRF, 2.66 [2.22]; HHC, 2.56 [2.12]) (Table 1).

Several characteristics for hospitals that discharged individuals with hip fracture to PAC differed by PAC setting before IPW, including number of beds (> 200 beds: SNF, 66.36%; IRF, 75.79%; HHC, 70.85%) and South region (SNF, 37.45%; IRF, 51.28%; HHC, 40.13%) (Table 2).

**Table 2:** Characteristics of hospitals where hip fracture hospitalizations occurred among individuals later receiving post-acute care, 2012–2018

**Characteristics**	**Overall** (*N* = 624,631)	**SNF** (*n* = 423,347)	**IRF** (*n* = 100,411)	**HHC** (*n* = 100,873)
Number of beds				
< 50 + Missing^a^	24,807 (3.98)	20,575 (4.86)	1,369 (1.36)	2,888 (2.87)
50–100	48,320 (7.75)	35,936 (8.49)	5,389 (5.37)	7,055 (7.00)
101–200	122,765 (19.68)	85,902 (20.29)	17,552 (17.48)	19,467 (19.30)
> 200	427,841 (68.59)	280,934 (66.36)	76,101 (75.79)	71,463 (70.85)
Medical school affiliation				
Major	123,538 (19.78)	85,306 (20.15)	18,092 (18.02)	20,140 (19.97)
Limited	117,496 (18.81)	77,889 (18.40)	20,801 (20.72)	18,806 (18.64)
Graduate	29,532 (4.73)	19,090 (4.51)	5,703 (5.68)	4,739 (4.70)
No Affiliation + Missing^a^	354,064 (56.68)	241,062 (56.94)	55,815 (55.59)	57,188 (56.69)
Urban/Rural				
Urban	523,639 (83.83)	346,416 (81.83)	88,109 (87.75)	89,114 (88.34)
Rural + Missing^a^	100,992 (16.17)	76,931 (18.17)	12,302 (12.25)	11,759 (11.66)
Region				
Northeast	118,521 (18.97)	83,398 (19.90)	19,659 (19.58)	15,464 (15.33)
Midwest	148,527 (23.78)	113,295 (26.80)	15,770 (15.71)	19,462 (19.29)
South	250,829 (40.16)	158,856 (37.45)	51,490 (51.28)	40,483 (40.13)
West	106,677 (17.08)	67,782 (15.83)	13,461 (13.41)	25,434 (25.21)
Other + Missing^a^	77 (0.01)	16 (0.00)	31 (0.03)	30 (0.03)
Ownership				
Government-owned	72,930 (11.68)	50,271 (11.89)	11,857 (11.81)	10,802 (10.71)
For-profit	90,264 (14.45)	55,005 (13.00)	19,546 (19.48)	15,713 (15.58)
Not-for-profit	447,290 (71.61)	308,313 (72.79)	66,716 (66.40)	72,261 (71.64)
Missing	14,147 (2.26)	9,758 (2.30)	2,292 (2.28)	2,097 (2.08)

Reports number (%)

*Abbreviations*: *HHC* Home Health Care, *IRF* Inpatient Rehabilitation Facilities, *SNF* Skilled Nursing Facilities

^a^Missing is not reported as a separate category due to data use agreements with the Centers for Medicare and Medicaid Services that prohibit the reporting of small cells (< 11 individuals)

### One-year cumulative incidences and incidence rates

The crude one-year cumulative incidences in the year after discharge from PAC were highest among individuals receiving IRF care for FRIs (SNF, 7.70%; IRF, 7.99%; HHC, 7.83%) and hospital readmission (SNF, 35.13%; IRF, 35.85%; HHC, 32.24%), but individuals receiving SNF care had the highest incidence of death (SNF, 10.66%; IRF, 3.65%; HHC, 4.92%) (Table 3). After IPW, the cumulative incidences were highest among individuals receiving HHC for FRIs, IRF care for hospital readmission, and SNF care for death (Additional file 1: Table S3). The most common FRIs were repeat hip fractures, followed by axial, lower extremity, and upper extremity fractures (Table 4, Additional file 1: Table S4).

**Table 3:** One-year outcomes after discharge from post-acute care between settings following hip fracture, 2012-2018

	**SNF** (*n* = 423,347)	**IRF** (*n* = 100,411)	**HHC** (*n* = 100,873)
**FRIs**			
Events, n	32,577	8,024	7,899
Crude cumulative incidence, %	7.70	7.99	7.83
Follow-up time, person-years	267,301	76,405	88,629
Crude incidence rate (95% CLs), per 1,000 person-years	123 (121, 123)	105 (102, 107)	89 (87, 91)
**Hospital Readmissions**			
Events, n	148,712	35,999	32,518
Crude cumulative incidence, %	35.13	35.85	32.24
Follow-up time, person-years	238,875	66,918	77,756
Crude incidence rate (95% CLs), per 1,000 person-years	623 (619, 626)	538 (532, 544)	418 (414, 423)
**Death**			
Events, n	45,142	3,665	4,963
Crude cumulative incidence, %	10.66	3.65	4.92
Follow-up time, person-years	270,301	77,451	90,620
Crude incidence rate (95% CLs), per 1,000 person-years	167 (165, 169)	47 (46, 49)	55 (53, 56)

Inverse probability of treatment weighted cumulative incidences and incidence rates are presented in Additional file 1: Table S3

*Abbreviations*: *CL* Confidence limits, *FRIs* Fall-related injuries, *HHC* Home Health Care, *IRF* Inpatient Rehabilitation Facilities, *SNF* Skilled Nursing Facilities

**Table 4:** One-year specific fall-related injury outcomes after discharge from post-acute care between settings following hip fracture, 2012-2018

	**SNF** (*n* = 423,347)	**IRF** (*n* = 100,411)	**HHC** (*n* = 100,873)
**Hip Fracture**			
Events, n	14,478	3,759	3,791
Crude cumulative incidence, %	3.42	3.74	3.76
Follow-up time, person-years	269,644	77,142	90,118
Crude incidence rate (95% CLs), per 1,000 person-years	54 (53, 55)	49 (47, 50)	42 (41, 43)
**Lower Extremity Fracture**			
Events, n	6,530	1,524	1,524
Crude cumulative incidence, %	1.54	1.52	1.51
Follow-up time, person-years	269,677	77,267	90,069
Crude incidence rate (95% CLs), per 1,000 person-years	24 (24, 25)	20 (19, 21)	17 (16, 18)
**Axial Fracture**			
Events, n	7,071	1,653	1,644
Crude cumulative incidence, %	1.67	1.65	1.63
Follow-up time, person-years	269,209	77,107	90,059
Crude incidence rate (95% CLs), per 1,000 person-years	26 (26, 27)	21 (20, 23)	18 (17, 19)
**Upper Extremity Fracture**			
Events, n	2,485	629	612
Crude cumulative incidence, %	0.59	0.63	0.61
Follow-up time, person-years	270,002	77,344	90,386
Crude incidence rate (95% CLs), per 1,000 person-years	9 (9, 10)	8 (8, 9)	7 (6, 7)
**Intracranial Bleeding**			
Events, n	1,953	493	452
Crude cumulative incidence, %	0.46	0.49	0.45
Follow-up time, person-years	267,000	77,359	90,472
Crude incidence rate (95% CLs), per 1,000 person-years	7 (7, 8)	6 (6, 7)	5 (5, 5)

Inverse probability of treatment weighted cumulative incidences and incidence rates are presented in Additional file 1: Table S4

*Abbreviations*: *CL* Confidence limits, *FRIs* Fall-related injuries, *HHC* Home Health Care, *IRF* Inpatient Rehabilitation Facilities, *SNF* Skilled Nursing Facilities

Crude IRs (95%CLs) per 1000 PYs in the year following discharge from PAC were highest among individuals receiving PAC in SNFs for FRIs (SNF, 123 [121, 123]; IRF, 105 [102, 107]; HHC, 89 [87, 91]), hospital readmission (SNF, 623 [619, 626]; IRF, 538 [532, 544]; HHC, 418 [414, 423]), and death (SNF, 167 [165, 169]; IRF, 47 [46,49]; HHC, 55 [53, 56]) (Table 3). After IPW, the IR for FRIs was highest among individuals receiving IRF care, while IRs for hospital readmission and death were highest among individuals receiving SNF care (Additional file 1: Table S3).

Crude and adjusted risk differences and rate differences for FRIs, hospital readmission, and death are presented in Additional file 1: Table S3. Crude and adjusted risk differences and rate differences for specific FRIs are presented in Additional file 1: Table S4.

### Risk ratios and hazard ratios

**Fig. 1:**
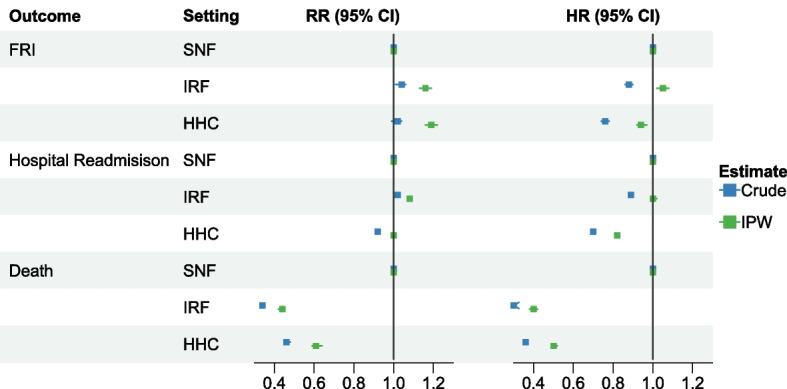
Associations between post-acute care setting following hip fracture and outcomes up to one year after discharge from post-acute care, 2012–2018. Presents risk ratios and hazard ratios before and after inverse probability of treatment weighting. Covariates included in adjusted models are listed in Additional file 1: Table S2. Abbreviations: CI, confidence interval; FRI, fall-related injuries; HHC, Home Health Care; HR, hazard ratio; IPW, inverse probability of treatment weighting; IRF, Inpatient Rehabilitation Facilities; RR, risk ratio; SNF, Skilled Nursing Facilities

**Fig. 2:**
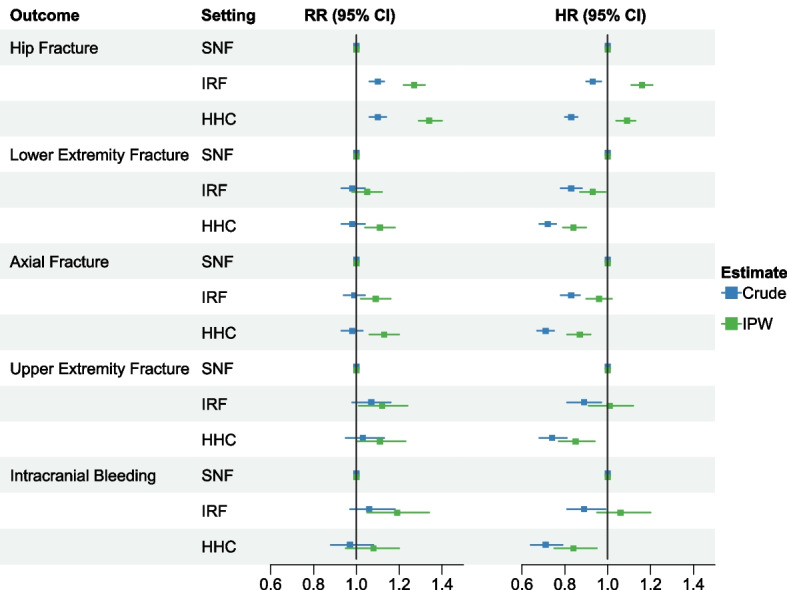
Associations between post-acute care setting following hip fracture and specific fall-related injury outcomes up to one year after discharge from post-acute care, 2012–2018. Presents risk ratios and hazard ratios before and after inverse probability of treatment weighting. Covariates included in adjusted models are listed in Additional file 1: Table S2. Abbreviations: CI, confidence interval; FRI, fall-related injuries; HHC, Home Health Care; HR, hazard ratio; IPW, inverse probability of treatment weighting; IRF, Inpatient Rehabilitation Facilities; RR, risk ratio; SNF, Skilled Nursing Facilities

After IPW, the risk of FRIs was 16% higher for individuals receiving IRF care and 19% higher for individuals receiving HHC care vs. SNF care (IRF vs. SNF, RR 1.16, 95%CLs 1.13, 1.19; HHC vs. SNF, RR 1.19, 95%CLs 1.16, 1.22) (Fig. 1, Additional file 1: Table S5). The risk of hospital readmission was 8% higher among IRF care recipients (RR 1.08; 95%CLs 1.07, 1.09) and similar for HHC recipients (RR 1.00; 95%CLs 0.99, 1.01) versus SNF care recipients. Both IRF and HHC recipients had a substantially lower risk of death after IPW versus SNF care recipients (IRF vs. SNF, RR 0.44, 95%CLs 0.42, 0.45; HHC vs. SNF, RR 0.61, 95%CLs 0.59, 0.64). The adjusted risk of hip fracture and axial fracture was higher among IRF care and HHC recipients versus SNF care (Fig. 2, Additional file 1: Table S5).

Compared to the SNF group, the rate of FRIs after IPW was 5% higher among the IRF group (HR 1.05; 95%CLs 1.02, 1.08) and 6% lower among the HHC group (HR 0.94; 95%CLs 0.92, 0.97) (Fig. 1, Additional file 1: Table S5). Recipients of HHC had an 18% lower rate of hospital readmission versus SNF care (HR 0.82; 95%CLs 0.81, 0.83) after IPW, but rates were similar for IRF versus SNF care (HR 1.00; 95%CLs 0.99, 1.02). The rate of death after IPW was 60% lower among IRF care recipients (HR 0.40; 95%CLs 0.38, 0.42) and 50% lower among HHC recipients (HR 0.50; 95%CLs 0.49, 0.52) versus SNF care recipients. Accounting for death as a competing risk, the rates of FRIs and hospital readmission were similar for IRF and SNF care recipients, while rates were lower for the HHC group versus SNF group (Additional file 1: Table S6). The rate of hip fracture was higher among individuals receiving IRF and HHC care versus SNF care after IPW (IRF vs. SNF, HR 1.16, 95%CLs 1.11, 1.21; HHC vs. SNF, HR 1.09, 95%CLs 1.04, 1.13) (Fig. 2, Additional file 1: Table S5). Adjusted rates of other specific FRIs were generally lower among the HHC group and not significantly different for the IRF group compared to the SNF group.

IPW adjusted survival curves for FRIs, hospital readmission, and death are presented in Additional file 1: Figures S2-4. Adjusted survival curves for specific FRIs are presented in Additional file 1: Figures S5-9.

### Sensitivity analysis

Across crude and IPW adjusted HRs, the lower confidence limits of the E-value ranged from 1.16 to 1.88 for FRIs, 1.00 to 1.85 for hospital readmission, and 3.26 to 5.91 for death (Additional file 1: Table S7). The lower confidence limits of E-values for crude and IPW adjusted RRs ranged from 1.00 to 1.59 for FRIs, 1.00 to 1.36 for hospital readmission, and 2.50 to 5.16 for death.

## Discussion

In this cohort of Medicare enrollees hospitalized for hip fracture, the crude rates of adverse outcomes were high in the year following PAC, especially among individuals who received PAC services in SNFs. In general, one-year rates of FRIs, hospital readmission, and death remained highest among SNF care recipients after covariate adjustment using inverse probability of treatment weighting and accounting for death as a competing risk. These results provide foundational knowledge that can inform future research efforts, quality improvement interventions, and policies aimed at improving outcomes for older adults receiving PAC for hip fracture.

Prior literature has examined the risk of subsequent fracture [19–22], rehospitalization [13, 16, 23–25], and mortality [4, 10, 16, 26–29] following hip fracture, although these studies began follow-up for outcomes at the time of hospital discharge or entry to PAC. Our study extends prior work by reporting one of the first estimates of adverse outcomes following discharge from PAC for hip fracture. Since PAC is intended to provide short-term care with the goal of facilitating recovery and successfully discharging patients to the community, understanding the risk of adverse outcomes after PAC discharge is meaningful for patients, clinicians, researchers, and policymakers. Our study also extends prior work by presenting both risk (using *persons* in the denominator) and rate (using *person-time* in the denominator) measures. For many outcomes, including FRIs and hospital readmissions, we observed that measures based on risk (e.g., risk ratios) and rates (e.g., hazard ratios) often led to different inferences.

We reported crude (unadjusted) risks and rates that provide needed foundational, descriptive epidemiological evidence on the nationwide burden of adverse outcomes in the year following PAC for hip fracture. We found that risks and rates of adverse outcomes were high for all PAC settings, although the rates of adverse outcomes were especially high for individuals receiving SNF care. Transitions of care services and closer follow-up with clinicians during the post-PAC period could improve outcomes for older adults with hip fracture, particularly those who received PAC in SNFs, although further examination is needed. It may be beneficial for future research to study the drivers of differences in outcomes across PAC settings, including delivery of services during PAC (e.g., duration and intensity of physical therapy, PAC quality of care), coordination of care with community providers after discharge from PAC, and payment models/incentives. Examining differences in adverse outcomes across high-risk subgroups (e.g., cognitive impairment, frailty) could help to target future interventions towards individuals who are most vulnerable. Additionally, reporting measures of post-PAC adverse outcomes using smaller geographic units (i.e., state, county) could be particularly informative for quality improvement interventions by clinicians, researchers, and policymakers at the local level.

Results comparing one-year adverse outcomes across PAC settings were exploratory, but can be used to inform future research efforts in this topic area. In advance of any future work to compare the effects of PAC settings on outcomes, it is worth questioning whether sufficient equipoise exists and the three PAC settings are actually exchangeable alternatives to one another. We found that the lower CLs for E-values were relatively small (< 1.60) for FRIs and hospital readmission after adjustment for 43 person- and hospital-level covariates, which suggests that a small to moderate amount of unmeasured confounding could shift estimates to the null. Future work could consider ascertaining additional covariate information to adjust for potential confounders, including factors at the patient-level (e.g., physical impairment, hip fracture severity, availability of caregivers to care for patients at home, out-of-pocket cost incurred by each setting) and system-level (e.g., availability of PAC services in a given geographic area [e.g., county], referral patterns to different PAC settings by hospitals, variation in PAC availability and referral patterns over time). However, differences in the characteristics of patients discharged to each PAC setting are likely so different that measuring and adjusting for all confounders would be extremely challenging. We also found that the group with greater FRIs and hospital readmission differed by measure (RR or HR), although the risks and rates of death were significantly higher among individuals receiving SNF care. Thus, researchers should strongly consider accounting for person-time under observation when studying the outcomes of PAC care, especially informative censoring by death. Finally, future work aiming to compare PAC settings should account for differences in length of stay. Since the average PAC length of stay differs between settings, starting follow-up at PAC discharge systematically covers different points in time relative to the hip fracture hospitalization.

### Limitations

Our study has several potential limitations. First, our results may not generalize well to individuals enrolled in Medicare Advantage, those without Medicare Part D, those who resided outside of the 48 contiguous states, had an FRI during PAC, or who received PAC in multiple settings. Second, our findings do not clarify the exact mechanisms by which differences in the services or care provided within each PAC setting might influence the observed differences in outcomes. We also do not know whether the observed differences in outcomes are true differential effects of the PAC setting after hip fracture, or if they instead represent residual confounding due to unmeasured covariates (e.g., functional status, social support). Residual confounding is probable and interpreting estimates as causal effects may be imprudent. Third, due to the nature of the data, we were not able to ascertain the exact day a person stopped receiving HHC services. Future research should re-estimate rates of adverse outcomes following receipt of HHC if this information becomes reliably available for all Medicare beneficiaries receiving HHC and intervention studies in the HHC setting should take into consideration the variation in the length of PAC. Fourth, the algorithm used to ascertain FRIs was initially developed for individuals receiving long-term care in nursing homes and requires further validation in the PAC setting [34].

Despite these limitations, we conducted a large, nationally representative study that provides some of the first estimates of adverse outcomes following discharge from hip fracture PAC in different settings. Our work also highlights the importance of calculating both risk and rate measures to assess the influence of differential time under observation across PAC groups.

## Conclusions

The risks and rates of FRIs, hospital readmission, and death were high following PAC for hip fracture. Crude risks and rates provided information on the nationwide burden of adverse outcomes in the year following PAC for hip fracture in the three most common PAC settings. Closer follow-up and additional services during the post-PAC period may help to improve outcomes for older adults with hip fracture, especially those who received PAC in SNFs, although further examination is needed. Our results suggest that future research should account for differing amounts of follow-up time across PAC settings and the presence of informative censoring by death.

## Supplementary Information

**Additional file 1: Figure S1.** Flow diagram of the study population. **Figure S2.** Inverse probability of treatment weighted one-year survival curves for fall-related injuries. **Figure S3.** Inverse probability of treatment weighted one-year survival curves for hospital readmission. **Figure S4.** Inverse probability of treatment weighted one-year survival curves for death. **Figure S5.** Inverse probability of treatment weighted one-year survival curves for hip fracture. **Figure S6.** Inverse probability of treatment weighted one-year survival curves for lower extremity fracture. **Figure S7.** Inverse probability of treatment weighted one-year survival curves for axial fracture. **Figure S8.** Inverse probability of treatment weighted one-year survival curves for upper extremity fracture. **Figure S9.** Inverse probability of treatment weighted one-year survival curves for intracranial bleeding. **Table S1.** International Classification of Diseases, Ninthand TenthRevision Codes Used to identify fall related injuries. **Table S2.** Standardized mean differences before and after inverse probability of treatment weighting among individuals discharged to different post-acute care settings after hip fracture hospitalization. **Table S3. **Risk differences and rate differences comparing one-year outcomes after discharge from post-acute care between settings following hip fracture, 2012-2018. **Table S4.** Risk differences and rate differences comparing one-year specific fall-related injury outcomes after discharge from post-acute care between settings following hip fracture, 2012-2018. **Table S5. **Associations between post-acute care setting following hip fracture and outcomes up to one year after discharge from post-acute care, 2012-2018. **Table S6. **Associations between post-acute care setting following hip fracture and outcomes, accounting for the competing risk of death. **Table S7.** E-values for quantitative bias sensitivity analyses.

## Data Availability

The data that support the findings of this study are available from the Centers for Medicare and Medicaid Services (CMS) but restrictions apply to the availability of these data, which were used under data use agreements for the current study, and so are not publicly available. However, other researchers can establish their own data use agreement and obtain the datasets employed through the Research Data Assistance Center (ResDAC), a CMS contractor that provides free assistance to researchers interested in CMS data.

## Abbreviations

CL: Confidence limit
FRI: Fall-related injury
HR: Hazard ratio
HHC: Home health care
IR: Incidence rate
IRF: Inpatient rehabilitation facility
IPW: Inverse-probability-of-treatment-weight
MBSF: Medicare Beneficiary Summary File
MedPAR: Medicare Provider Analysis and Review
MDS: Minimum Data Set
OASIS: Outcome and Assessment Information Set
PY: Person-year
PAC: Post-acute care
RR: Risk ratio
SNF: Skilled nursing facility
SD: Standard deviation
