# Supplementary material for: Evaluation of post-acute care and one-year outcomes among Medicare beneficiaries with hip fractures: a retrospective cohort study
Source: BMC Med. 2023 Jul 3;21:232. doi: 10.1186/s12916-023-02958-9 (PMC10318833; doi:10.1186/s12916-023-02958-9)
Supplement: Supplementary file 1 — Additional file 1: Figure S1. Flow diagram of the study population. Figure S2. Inverse probability of treatment weighted one-year survival curves for fall-related injuries. Figure S3. Inverse probability of treatment weighted one-year survival curves for hospital readmission. Figure S4. Inverse probability of treatment weighted one-year survival curves for death. Figure S5. Inverse probability of treatment weighted one-year survival curves for hip fracture. Figure S6. Inverse probability of treatment weighted one-year survival curves for lower extremity fracture. Figure S7. Inverse probability of treatment weighted one-year survival curves for axial fracture. Figure S8. Inverse probability of treatment weighted one-year survival curves for upper extremity fracture. Figure S9. Inverse probability of treatment weighted one-year survival curves for intracranial bleeding. Table S1. International Classification of Diseases, Ninthand TenthRevision Codes Used to identify fall related injuries. Table S2. Standardized mean differences before and after inverse probability of treatment weighting among individuals discharged to different post-acute care settings after hip fracture hospitalization. Table S3. Risk differences and rate differences comparing one-year outcomes after discharge from post-acute care between settings following hip fracture, 2012-2018. Table S4. Risk differences and rate differences comparing one-year specific fall-related injury outcomes after discharge from post-acute care between settings following hip fracture, 2012-2018. Table S5. Associations between post-acute care setting following hip fracture and outcomes up to one year after discharge from post-acute care, 2012-2018. Table S6. Associations between post-acute care setting following hip fracture and outcomes, accounting for the competing risk of death. Table S7. E-values for quantitative bias sensitivity analyses. [file 12916_2023_2958_MOESM1_ESM.docx]

**Additional File 1**

**Title**: Evaluation of Post-Acute Care and One-year Outcomes Among Medicare Beneficiaries with Hip Fractures: A Retrospective Cohort Study

**Authors**: Melissa R. Riester, PharmD, Francesca L. Beaudoin, MD, PhD, Richa Joshi, MS, MBA, Kaleen N. Hayes, PharmD, PhD, Meghan A. Cupp, MPH, Sarah D. Berry, MD, MPH, Andrew R. Zullo, PharmD, PhD

**Figure S1**. Flow Diagram of the Study Population.

**Figure S2**. Inverse Probability of Treatment Weighted One-Year Survival Curves for Fall-Related Injuries.

**Figure S3**. Inverse Probability of Treatment Weighted One-Year Survival Curves for Hospital Readmission.

**Figure S4**. Inverse Probability of Treatment Weighted One-Year Survival Curves for Death.

**Figure S5**. Inverse Probability of Treatment Weighted One-Year Survival Curves for Hip Fracture.

**Figure S6**. Inverse Probability of Treatment Weighted One-Year Survival Curves for Lower Extremity Fracture.

**Figure S7**. Inverse Probability of Treatment Weighted One-Year Survival Curves for Axial Fracture.

**Figure S8**. Inverse Probability of Treatment Weighted One-Year Survival Curves for Upper Extremity Fracture.

**Figure S9**. Inverse Probability of Treatment Weighted One-Year Survival Curves for Intracranial Bleeding.

**Table S1**. International Classification of Diseases, Ninth (ICD-9) and Tenth (ICD-10) Revision Codes Used to Identify Fall Related Injuries.

**Table S2**. Standardized Mean Differences Before and After Inverse Probability of Treatment Weighting Among Individuals Discharged to Different Post-Acute Care Settings After Hip Fracture Hospitalization.

**Table S3.** Risk Differences and Rate Differences Comparing One-year Outcomes after Discharge from Post-Acute Care between Settings Following Hip Fracture, 2012-2018.

**Table S4**. Risk Differences and Rate Differences Comparing One-year Specific Fall-Related Injury Outcomes after Discharge from Post-Acute Care between Settings following Hip Fracture, 2012-2018.

**Table S5.** Associations Between Post-Acute Care Setting Following Hip Fracture and Outcomes up to One Year after Discharge from Post-Acute Care, 2012-2018.

**Table S6.** Associations Between Post-Acute Care Setting Following Hip Fracture and Outcomes, Accounting for the Competing Risk of Death.

**Table S7**. E-values for quantitative bias sensitivity analyses.

**Figure S1. Flow Diagram of the Study Population.**


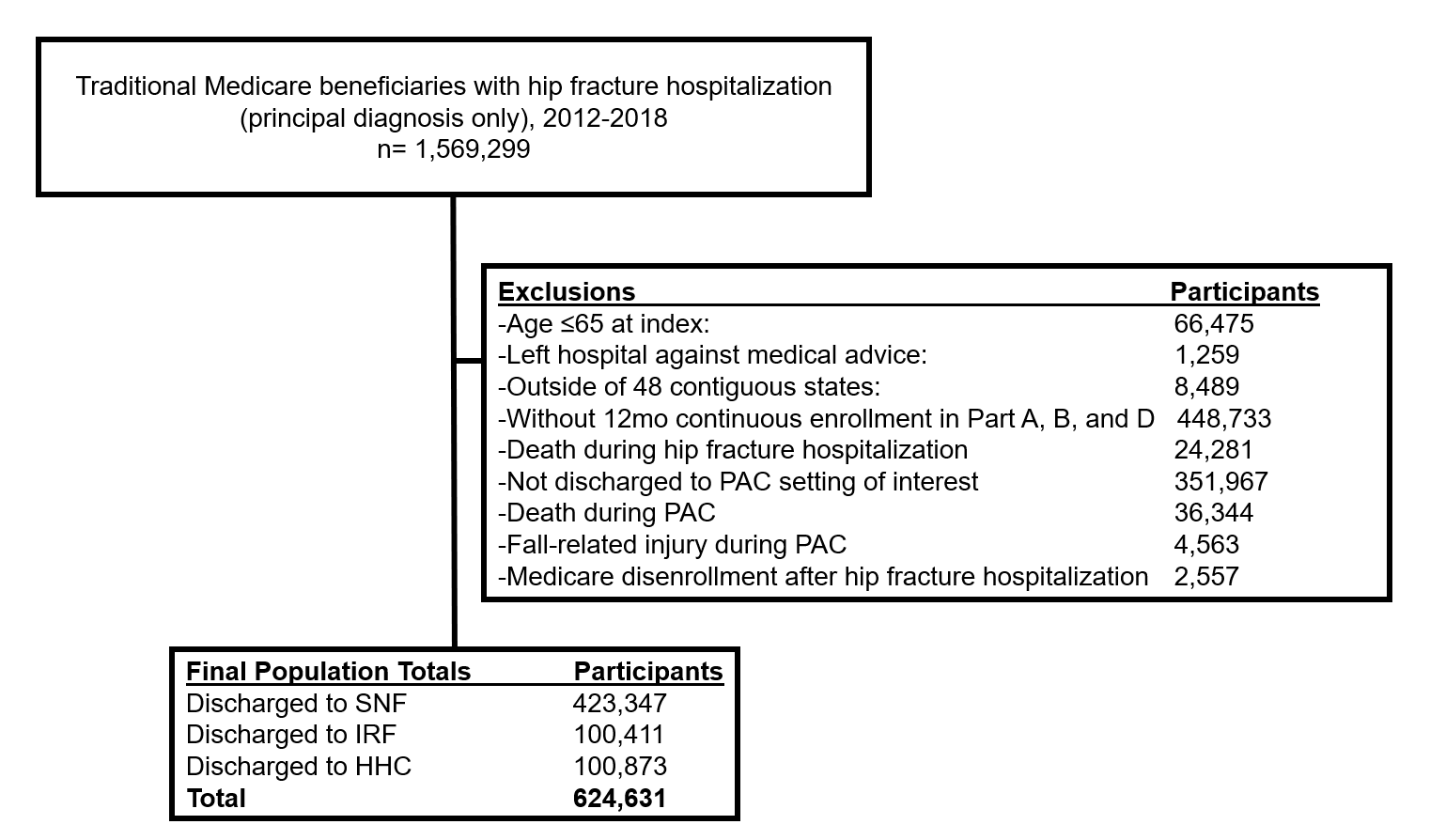


**Abbreviations:** HHC, Home Health Care; IRF, Inpatient Rehabilitation Facilities; mo, months; PAC, post-acute care; SNF, Skilled Nursing Facilities.

**Figure S2. Inverse Probability of Treatment Weighted One-Year Survival Curves for Fall-Related Injuries.**


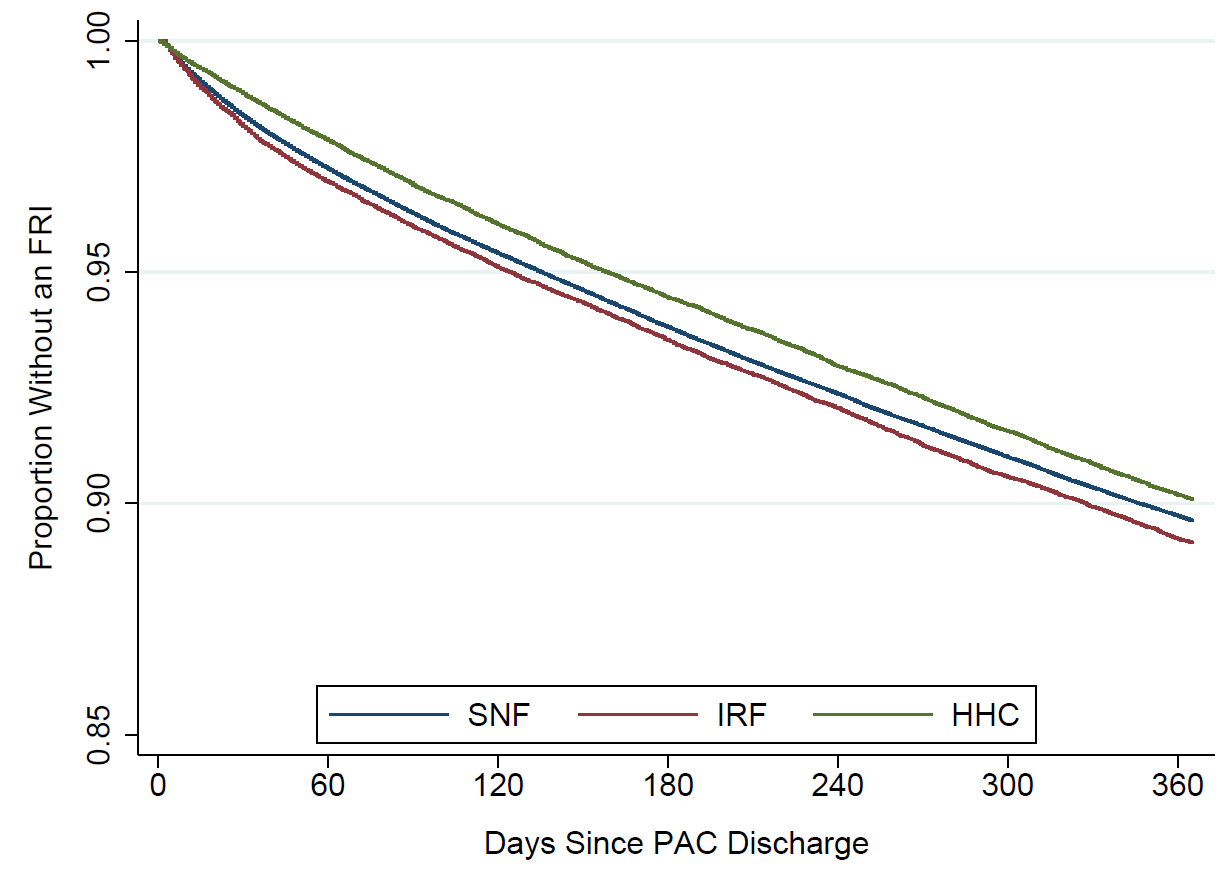


**Abbreviations**: FRI, fall-related injury; HHC, Home Health Care; IRF, Inpatient Rehabilitation Facilities; PAC, post-acute care; SNF, Skilled Nursing Facilities.

**Figure S3. Inverse Probability of Treatment Weighted One-Year Survival Curves for Hospital Readmission.**


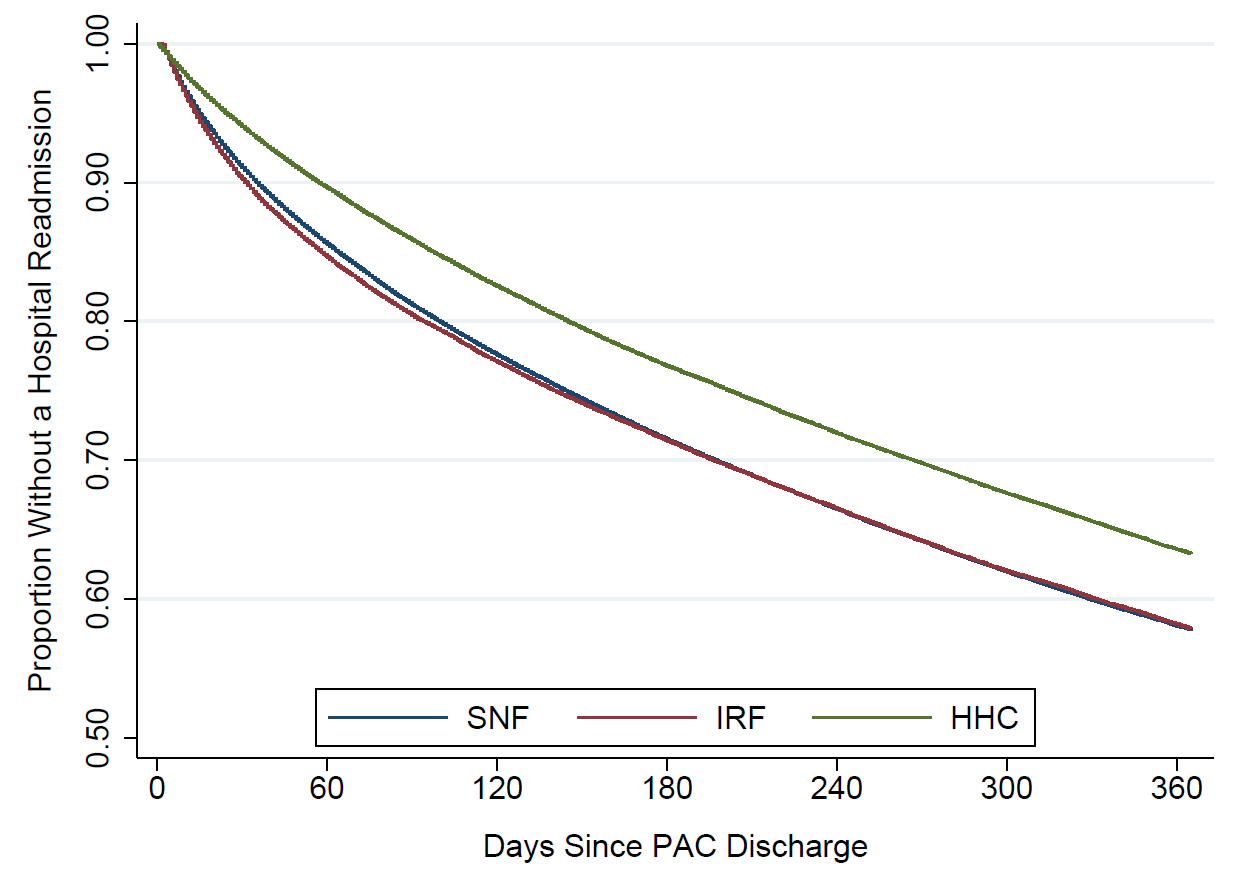


**Abbreviations:** HHC, Home Health Care; IRF, Inpatient Rehabilitation Facilities; PAC, post-acute care; SNF, Skilled Nursing Facilities.

**Figure S4. Inverse Probability of Treatment Weighted One-Year Survival Curves for Death.**

**
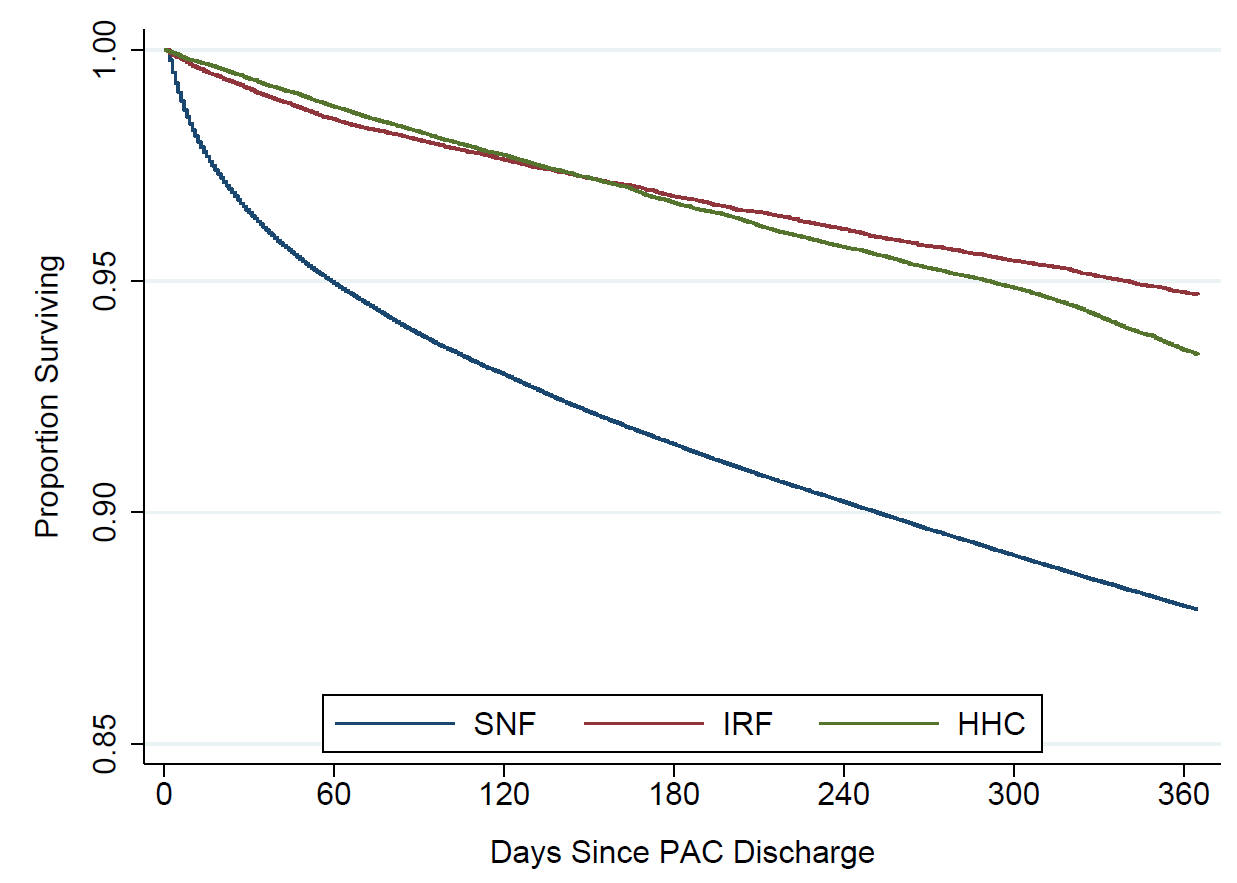
**

**Abbreviations:** HHC, Home Health Care; IRF, Inpatient Rehabilitation Facilities; PAC, post-acute care; SNF, Skilled Nursing Facilities.

**Figure S5. Inverse Probability of Treatment Weighted One-Year Survival Curves for Hip Fracture.**

**
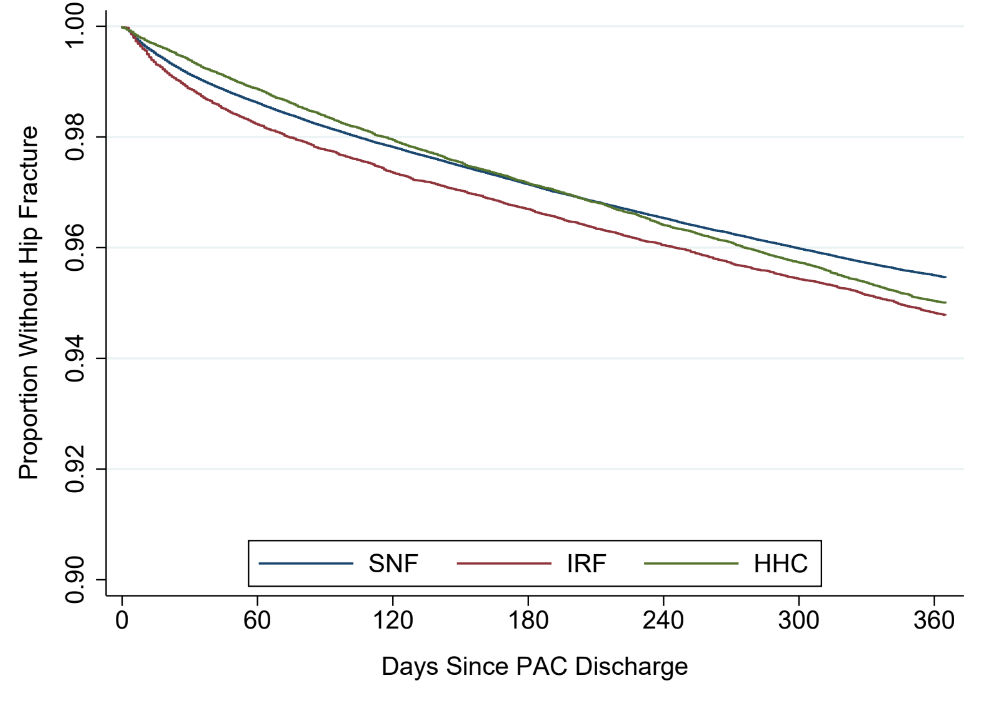
**

**Abbreviations:** HHC, Home Health Care; IRF, Inpatient Rehabilitation Facilities; PAC, post-acute care; SNF, Skilled Nursing Facilities.

**Figure S6. Inverse Probability of Treatment Weighted One-Year Survival Curves for Lower Extremity Fracture.**

**
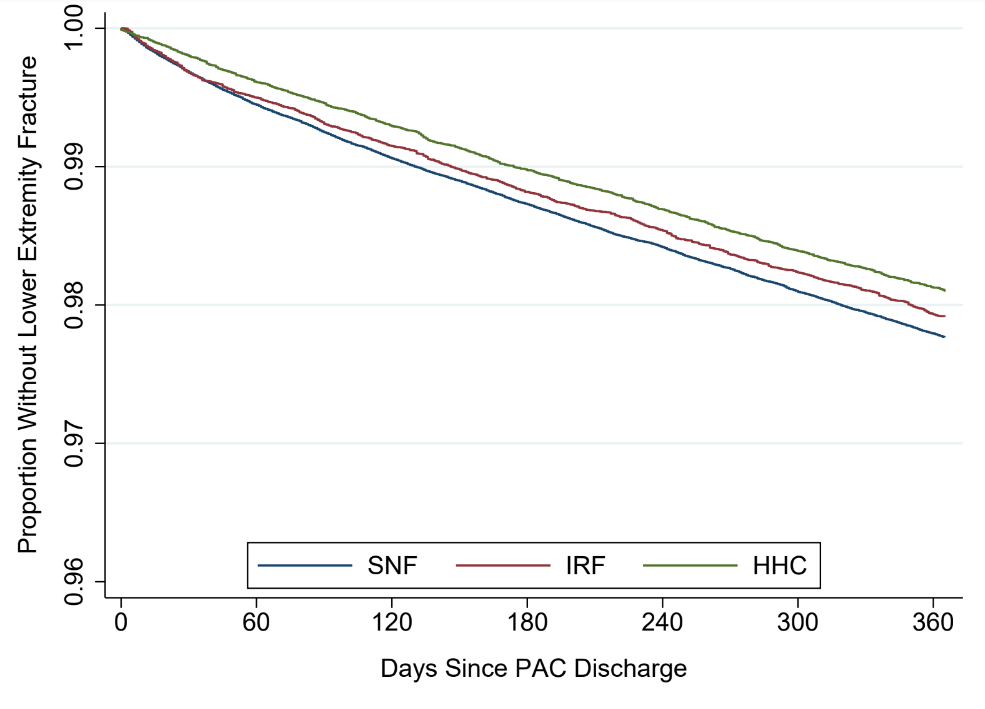
**

**Abbreviations:** HHC, Home Health Care; IRF, Inpatient Rehabilitation Facilities; PAC, post-acute care; SNF, Skilled Nursing Facilities.

**Figure S7. Inverse Probability of Treatment Weighted One-Year Survival Curves for Axial Fracture.**

**
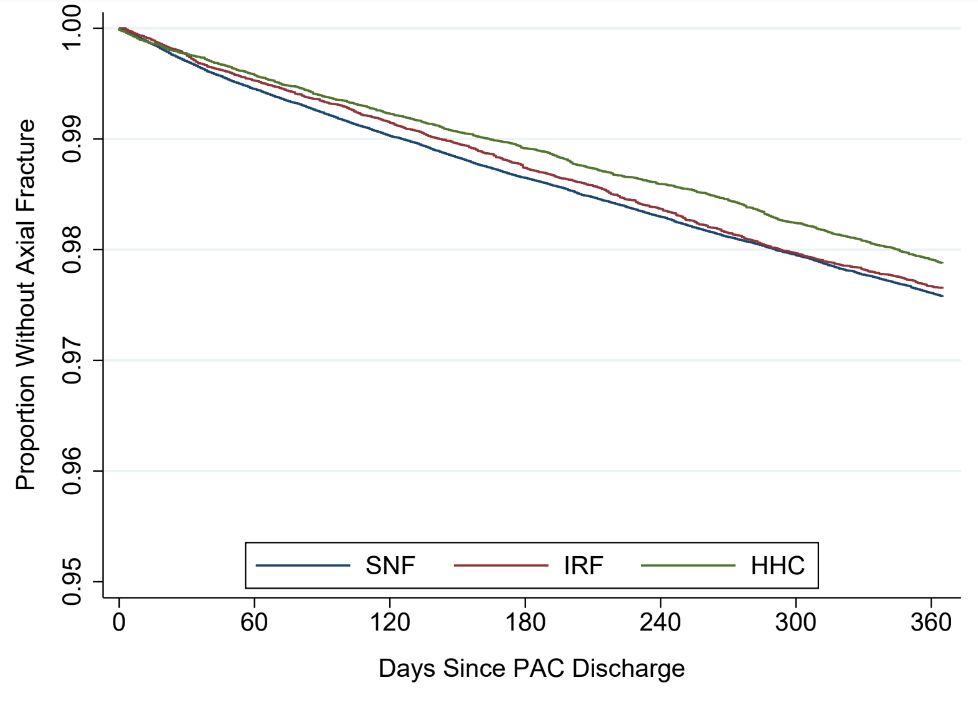
**

**Abbreviations:** HHC, Home Health Care; IRF, Inpatient Rehabilitation Facilities; PAC, post-acute care; SNF, Skilled Nursing Facilities.

**Figure S8. Inverse Probability of Treatment Weighted One-Year Survival Curves for Upper Extremity Fracture.**

**
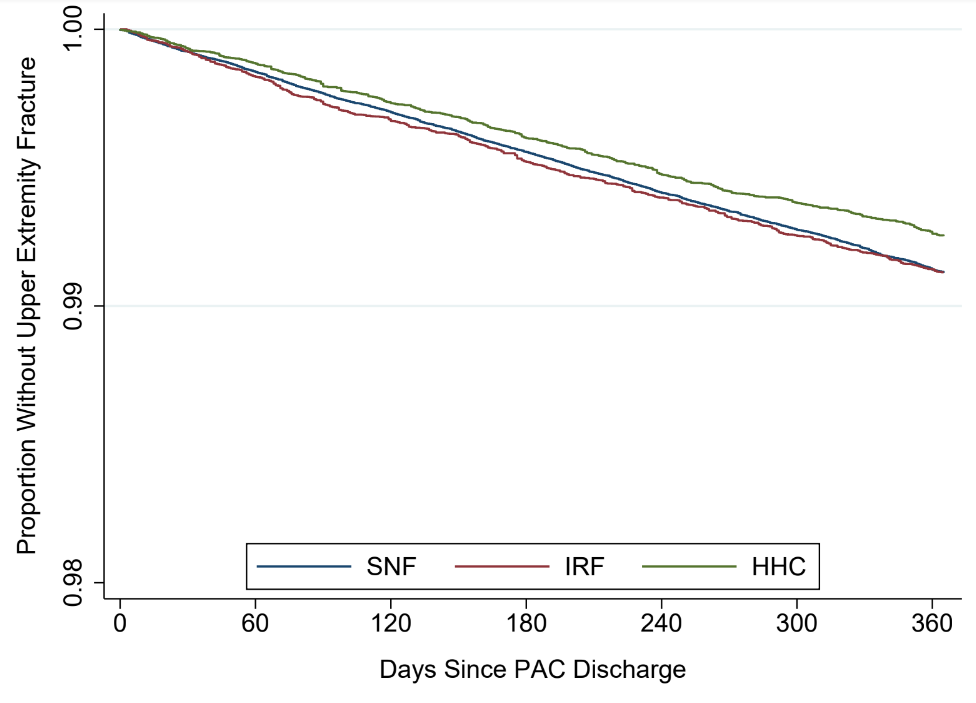
**

**Abbreviations:** HHC, Home Health Care; IRF, Inpatient Rehabilitation Facilities; PAC, post-acute care; SNF, Skilled Nursing Facilities.

**Figure S9. Inverse Probability of Treatment Weighted One-Year Survival Curves for Intracranial Bleeding.**

**
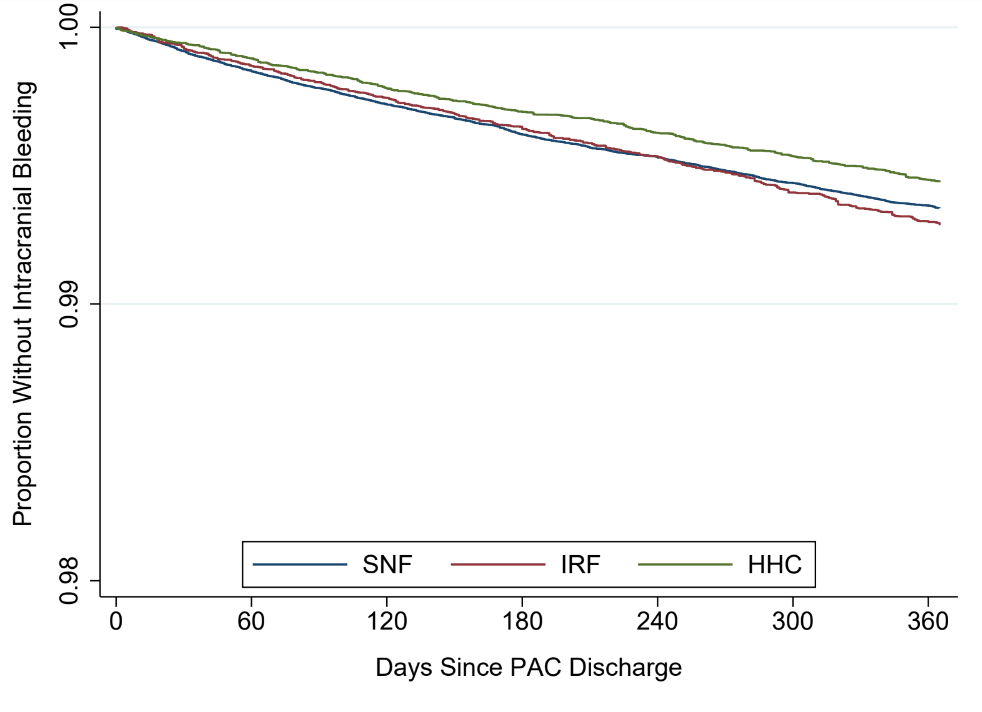
**

**Abbreviations:** HHC, Home Health Care; IRF, Inpatient Rehabilitation Facilities; PAC, post-acute care; SNF, Skilled Nursing Facilities.

**Table S1. International Classification of Diseases, Ninth (ICD-9) and Tenth (ICD-10) Revision Codes Used to Identify Fall Related Injuries.**

| **ICD-9 Diagnosis Codes** | **ICD-10 Diagnosis Codes** |
| --- | --- |
| **Fall-Related Injury** | |
| Composite of the individual injuries listed below | |
| **Hip Fracture** | |
| 820.xx, 733.14 | S72.0XXX, S72.1XXX, M80.05XX, M84.45XX, M80.85XX, M84.65XX |
| **Lower Extremity Fracture** | |
| 821.xx | S72.2XXX, S72.3XXX, S72.4XXX, S72.8XXX, S72.9XXX |
| 822.xx | S82.0XXX |
| 823.xx | S82.1XXX, S82.2XXX, S82.4XXX, S82.8XXX, S82.9XXX, M80.06XX, M84.46XX, M80.86XX, M84.66XX |
| 824.xx | S82.3XXX, S82.5XXX, S82.6XXX, M80.07XX, M84.471X-M84.473X, M84.371X-M84.373X, M84.471X-M84.473X, M84.671X-M84.673X |
| 808.xx | S32.1XXX, S32.2XXX, S33.3XXX, S32.4XXX, S32.5XXX, S32.6XXX, S32.8XXX, S32.9XXX, M48.57XX-M48.58XX |
| **Axial Fracture** | |
| 807.0x-807.1x, 807.2-807.4 | S22.2XXX, S22.3XXX, S22.4XXX, S22.5XXX, S22.9XXX |
| 810.xx, 811.xx | S42.0XXX, S42.1XXX, S42.9XXX, M80.01XX, M84.41XX, M80.81XX, M84.61XX |
| 805.xx, 806.xx | S12.XXXX, S14.0XXX, M48.51XX-M48.53XX |
| 805.xx, 806.xx, 733.13 | S22.0XXX, S32.0XXX, S24.0XXX, S34.0XXX, M80.08XX, M80.88XX, M48.50XX, M48.54XX-M48.56XX |
| **Upper Extremity Fracture** | |
| 812.xx | S42.2XXX, S42.3XXX, S42.4XXX, M80.02XX, M84.42XX, M80.82XX, M84.62XX |
| 813.xx | S52.0XXX, S52.1XXX, S52.2XXX, S53.3XXX, S52.5XXX, S52.6XXX, S52.9XXX, M84.43XX, M80.83XX, M84.63XX |
| 814.00-814.19 | S62.0XXX, S62.1XXX, S62.9XXX |
| **Intracranial Bleeding** | |
| 851.xx, 852.xx, 853.xx | S06.1XXX, S06.2XXX, S06.3XXX, S06.4XXX, S06.5XXX, S06.6XXX, S06.9XXX |

**Table S2. Standardized Mean Differences Before and After Inverse Probability of Treatment Weighting Among Individuals Discharged to Different Post-Acute Care Settings After Hip Fracture Hospitalization**.

|  | **SMDs** | | | | | |
| --- | --- | --- | --- | --- | --- | --- |
|  | **IRF vs. SNF** | | **HHC vs. SNF** | | **HHC vs. IRF** | |
|  | **Crude** | **IPW** | **Crude** | **IPW** | **Crude** | **IPW** |
| **Characteristics** | | | | | | |
| Age, years | -0.39 | -0.02 | -0.423 | 0.00 | -0.04 | 0.02 |
| Sex | -0.08 | -0.01 | -0.072 | 0.00 | 0.01 | 0.01 |
| Race/ethnicity | 0.04 | 0.01 | 0.065 | 0.01 | 0.02 | 0.01 |
| Dual Medicare/Medicaid enrollment | -0.25 | -0.02 | -0.252 | 0.01 | -0.00 | 0.03 |
| Calendar year of fracture | -0.08 | -0.02 | 0.017 | 0.00 | 0.09 | 0.02 |
| **Conditions**^a^ | | | | | | |
| Anemia | 0.02 | 0.01 | -0.106 | 0.01 | -0.13 | -0.01 |
| Cancer | 0.04 | 0.01 | -0.014 | 0.01 | -0.06 | -0.00 |
| Cardiac dysrhythmias | -0.01 | 0.02 | -0.115 | 0.00 | -0.10 | -0.02 |
| Cerebrovascular disease | -0.01 | 0.01 | -0.048 | 0.01 | -0.04 | -0.00 |
| Chronic kidney disease | -0.03 | -0.00 | -0.032 | 0.00 | -0.00 | 0.01 |
| Coronary atherosclerosis and other heart disease | -0.01 | -0.00 | -0.033 | 0.00 | -0.02 | 0.00 |
| Dementia, delirium, and other cognitive disorders | -0.37 | -0.01 | -0.28 | 0.00 | 0.09 | 0.01 |
| Diabetes mellitus | 0.03 | 0.01 | -0.021 | 0.00 | -0.05 | -0.00 |
| Heart valve disorders | 0.00 | 0.01 | -0.075 | 0.00 | -0.08 | -0.01 |
| Hypertension | 0.06 | 0.02 | -0.051 | 0.00 | -0.11 | -0.02 |
| Low back pain | 0.01 | 0.00 | 0.025 | 0.00 | 0.02 | -0.00 |
| Obesity | 0.03 | 0.00 | 0.009 | 0.00 | -0.02 | -0.00 |
| Opioid related disorders | -0.03 | -0.00 | -0.015 | 0.00 | 0.02 | 0.01 |
| Osteoarthritis | 0.03 | 0.01 | -0.013 | -0.01 | -0.05 | -0.02 |
| Peripheral and visceral atherosclerosis | -0.01 | 0.00 | 0.026 | -0.01 | 0.04 | -0.01 |
| Phlebitis, thrombophlebitis, and thromboembolism | 0.01 | 0.01 | -0.052 | 0.01 | -0.06 | -0.00 |
| Pulmonary heart disease | -0.01 | 0.01 | -0.064 | 0.00 | -0.05 | -0.01 |
| Rheumatoid arthritis and related disease | 0.01 | 0.00 | 0.008 | 0.00 | -0.00 | 0.01 |
| Schizophrenia and other psychotic disorders | -0.06 | 0.00 | -0.075 | 0.00 | -0.01 | 0.00 |
| Thyroid disorder | 0.00 | 0.00 | -0.061 | 0.00 | -0.07 | -0.00 |
| Frailty index^b^ | 0.34 | 0.01 | 0.389 | -0.00 | 0.06 | -0.02 |
| Gagne comorbidity score^c^ | -0.43 | -0.02 | -0.45 | 0.00 | -0.03 | 0.02 |
| **Medication use before the hip fracture hospitalization**^d^ | | | | | | |
| Opioids | -0.08 | 0.01 | -0.08 | 0.00 | 0.00 | -0.00 |
| NSAIDs | -0.01 | 0.01 | -0.00 | 0.00 | 0.01 | -0.00 |
| Gabapentinoids | -0.07 | 0.01 | -0.07 | 0.00 | 0.00 | -0.00 |
| Benzodiazepines | -0.11 | 0.01 | -0.09 | 0.00 | 0.02 | -0.00 |
| **Hip fracture hospitalization characteristics** | | | | | | |
| Intensive care unit use during hip fracture hospitalization | 0.01 | 0.02 | -0.10 | 0.01 | -0.11 | -0.01 |
| Length of stay | -0.14 | 0.03 | -0.11 | 0.03 | 0.02 | -0.00 |
| Hospital complications |  |  |  |  |  |  |
| Urinary tract infections | -0.15 | -0.01 | -0.16 | 0.01 | -0.01 | 0.02 |
| Pressure ulcer of skin | -0.05 | 0.00 | -0.04 | 0.01 | 0.01 | 0.01 |
| Pneumonia | -0.06 | 0.01 | -0.06 | 0.00 | -0.01 | -0.00 |
| Fracture management |  |  |  |  |  |  |
| Partial or total joint replacement | 0.10 | 0.01 | 0.03 | -0.01 | -0.07 | -0.01 |
| Internal fixation (any) or external fixation (open or percutaneous approach) | -0.03 | 0.01 | -0.02 | 0.00 | 0.02 | -0.00 |
| Non-surgical management | -0.05 | 0.01 | -0.13 | 0.01 | -0.07 | 0.00 |
| **Hospital Characteristics** | | | | | | |
| For-profit | 0.18 | 0.02 | 0.07 | 0.00 | -0.10 | -0.01 |
| Not-for-profit | -0.14 | -0.01 | -0.03 | -0.01 | 0.11 | -0.00 |
| Government-owned | -0.00 | -0.01 | -0.04 | 0.01 | -0.04 | 0.01 |
| Region | -0.17 | -0.01 | 0.10 | 0.01 | 0.26 | 0.02 |
| **Abbreviations:** HHC, Home Health Care; IPW, inverse probability of treatment weighting; IRF, Inpatient Rehabilitation Facilities; NSAIDs, Non-Steroidal Anti-Inflammatory Drugs; SMDs, standardized mean differences; SNF, Skilled Nursing Facilities.  ^a^Represents the conditions documented on the hip fracture hospitalization claim.  ^b^Measured using the Claims-based Frailty Index and categorized as: <0.15 (robust), 0.15-0.24 (prefrail), ≥0.25 (mildly-to-severely frail).  ^d^Measured using the Gagne Combined Comorbidity Score, ranging from -2 to 26, where higher scores indicate greater multimorbidity.  ^d^Medication use was defined as at least one dispensing in the 12 months prior to the hip fracture hospitalization. | | | | | | |

**Table S3. Risk Differences and Rate Differences Comparing One-year Outcomes after Discharge from Post-Acute Care between Settings Following Hip Fracture, 2012-2018.**

| **Setting^a^** | **Crude Risk Difference, % (95% CLs)** | **Crude Rate Difference (95% CLs) per 1,000 PYs** | **IPW Cumulative Incidence, % (95% CLs)** | **IPW Risk Difference, % (95% CLs)** | **IPW Rates (95% CLs) per 1,000 PYs** | **IPW Rate Difference (95% CLs) per 1,000 PYs** |
| --- | --- | --- | --- | --- | --- | --- |
| **FRIs** | | | | | | |
| SNF | Reference | Reference | 7.55 (7.47, 7.64) | Reference | 116 (115, 117) | Reference |
| IRF | 0.29 (0.11, 0.48) | -17 (-20, -14) | 8.78 (8.56, 9.00) | 1.23 (0.99, 1.46) | 120 (117, 123) | 4 (0, 8) |
| HHC | 0.13 (-0.05, 0.32) | -33 (-35, -30) | 9.00 (8.78, 9.22) | 1.45 (1.21, 1.68) | 106 (104, 109) | -10 (-13, -7) |
| **Hospital Readmission** | | | | | | |
| SNF | Reference | Reference | 35.09 (34.94, 35.24) | Reference | 602 (598, 605) | Reference |
| IRF | 0.72 (0.39,1.05) | -85 (-91, -78) | 37.81 (37.44, 38.18) | 2.72 (2.32, 3.12) | 594 (586, 601) | -8 (-17, 0) |
| HHC | -2.89 (-3.21, -2.57) | -204 (-210, -199) | 34.99 (34.64, 35.34) | -0.10 (-0.49, 0.28) | 476 (470, 482) | -126 (-133, -119) |
| **Death** | | | | | | |
| SNF | Reference | Reference | 9.63 (9.54, 9.72) | Reference | 145 (143, 146) | Reference |
| IRF | -7.01 (-7.16, -6.87) | -120 (-122, -117) | 4.19 (4.02, 4.36) | -5.44 (-5.64, -5.26) | 57 (54, 59) | -88 (-91, -86) |
| HHC | -5.74 (-5.91, -5.58) | -112 (-114, -110) | 5.92 (5.73, 6.11) | -3.71 (-3.92, -3.51) | 68 (66, 70) | -77 (-79, -74) |
| **Abbreviations:** CL, confidence limits; FRIs, fall-related injuries; HHC, Home Health Care; IPW, inverse probability of treatment weighted; IRF, Inpatient Rehabilitation Facilities; PYs, person-years; SNF, Skilled Nursing Facilities.  **Note:** Crude cumulative incidences and incidence rates are presented in Table 3.  ^a^Number of participants: n= 423,347 (SNF), n= 100,411 (IRF), n= 100,873 (HHC). | | | | | | |

**Table S4. Risk Differences and Rate Differences Comparing One-year Specific Fall-Related Injury Outcomes after Discharge from Post-Acute Care between Settings following Hip Fracture, 2012-2018.**

| **Setting^a^** | **Crude Risk Difference, % (95% CLs)** | **Crude Rate Difference (95% CLs) per 1,000 PYs** | **IPW Cumulative Incidence, % (95% CLs)** | **IPW Risk Difference, % (95% CLs)** | **IPW Rates (95% CLs) per 1,000 PYs** | **IPW Rate Difference (95% CLs) per 1,000 PYs** |
| --- | --- | --- | --- | --- | --- | --- |
| **Hip Fracture** | | | | | | |
| SNF | Reference | Reference | 3.30 (3.25, 3.36) | Reference | 50 (49, 51) | Reference |
| IRF | 0.32 (0.19, 0.45) | -5 (-7, -3) | 4.19 (4.03, 4.36) | 0.89 (0.72, 1.06) | 57 (55, 59) | 7 (5, 9) |
| HHC | 0.34 (0.20, 0.46) | -12 (-9, -5) | 4.43 (4.27, 4.59) | 1.13 (0.96, 1.30) | 52 (50, 54) | 2 (0, 4) |
| **Lower Extremity Fracture** | | | | | | |
| SNF | Reference | Reference | 1.54 (1.50, 1.57) | Reference | 24 (23, 24) | Reference |
| IRF | -0.02 (-0.11, 0.06) | -4 (-6, -3) | 1.61 (1.51, 1.71) | 0.07 (-0.03, 0.18) | 22 (20, 23) | -2 (-3, 0) |
| HHC | -0.03 (-0.12, 0.05) | -7 (-8, -6) | 1.70 (1.60, 1.80) | 0.16 (0.06, 0.27) | 19 (18, 21) | -4 (-6, -3) |
| **Axial Fracture** | | | | | | |
| SNF | Reference | Reference | 1.66 (1.62, 1.70) | Reference | 26 (25, 26) | Reference |
| IRF | -0.02 (-0.11, 0.06) | -5 (-6, -4) | 1.81 (1.71, 1.92) | 0.15 (0.04, 0.26) | 24 (23, 26) | -1 (-3, 0) |
| HHC | -0.04 (-0.13, 0.05) | -3 (-5, -2) | 1.88 (1.77, 1.99) | 0.22 (0.10, 0.33) | 22 (20, 23) | -4 (-5, -2) |
| **Upper Extremity Fracture** | | | | | | |
| SNF | Reference | Reference | 0.59 (0.56, 0.61) | Reference | 9 (9, 9) | Reference |
| IRF | 0.04 (-0.02, 0.09) | -1 (-2, 0) | 0.66 (0.60, 0.72) | 0.07 (0.01, 0.14) | 9 (8, 10) | 0 (0, 0) |
| HHC | 0.02 (-0.03, 0.07) | -2 (-3, -2) | 0.65 (0.59, 0.72) | 0.06 (0.00, 0.13) | 8 (7, 8) | -1 (-2, 0) |
| **Intracranial Bleeding** | | | | | | |
| SNF | Reference | Reference | 0.45 (0.43, 0.47) | Reference | 7 (7, 7) | Reference |
| IRF | 0.03 (-0.02, 0.08) | -1 (-2, 0) | 0.54 (0.48, 0.60) | 0.09 (0.03, 0.15) | 7 (7, 8) | 0 (0, 1) |
| HHC | -0.01 (-0.06, 0.03) | -2 (-3, -2) | 0.48 (0.43, 0.54) | 0.03 (-0.02, 0.09) | 5 (5, 6) | -1 (-2, 0) |
| **Abbreviations**: CL, confidence limits; FRIs, fall-related injuries; HHC, Home Health Care; IPW, inverse probability of treatment weighted; IRF, Inpatient Rehabilitation Facilities; PYs, person-years; SNF, Skilled Nursing Facilities.  **Note:** Crude cumulative incidences and incidence rates are presented in Table 4.  ^a^Number of participants: n= 423,347 (SNF), n= 100,411 (IRF), n= 100,873 (HHC). | | | | | | |

**Table S5. Associations Between Post-Acute Care Setting Following Hip Fracture and Outcomes up to One Year after Discharge from Post-Acute Care, 2012-2018.**

| **Setting** | **Crude Risk Ratio (95% CLs)** | **IPW Risk Ratio (95% CLs)** | **Crude Hazard Ratio (95% CLs)** | **IPW Hazard Ratio (95% CLs)** |
| --- | --- | --- | --- | --- |
| **FRIs** | | | | |
| SNF | Reference | Reference | Reference | Reference |
| IRF | 1.04 (1.01, 1.06) | 1.16 (1.13, 1.19) | 0.88 (0.86, 0.90) | 1.05 (1.02, 1.08) |
| HHC | 1.02 (0.99, 1.04) | 1.19 (1.16, 1.22) | 0.76 (0.74, 0.78) | 0.94 (0.92, 0.97) |
| **Hospital Readmission** | | | | |
| SNF | Reference | Reference | Reference | Reference |
| IRF | 1.02 (1.01, 1.03) | 1.08 (1.07, 1.09) | 0.89 (0.88, 0.90) | 1.00 (0.99, 1.02) |
| HHC | 0.92 (0.91, 0.93) | 1.00 (0.99, 1.01) | 0.70 (0.70, 0.71) | 0.82 (0.81, 0.83) |
| **Death** | | | | |
| SNF | Reference | Reference | Reference | Reference |
| IRF | 0.34 (0.33, 0.35) | 0.44 (0.42, 0.45) | 0.30 (0.29, 0.31) | 0.40 (0.38, 0.42) |
| HHC | 0.46 (0.45, 0.48) | 0.61 (0.59, 0.64) | 0.36 (0.35, 0.37) | 0.50 (0.49, 0.52) |
| **Hip Fracture** | | | | |
| SNF | Reference | Reference | Reference | Reference |
| IRF | 1.10 (1.06, 1.13) | 1.27 (1.22, 1.32) | 0.93 (0.90, 0.97) | 1.16 (1.11, 1.21) |
| HHC | 1.10 (1.06, 1.14) | 1.34 (1.29, 1.40) | 0.83 (0.80, 0.86) | 1.09 (1.04, 1.13) |
| **Lower Extremity Fracture** | | | | |
| SNF | Reference | Reference | Reference | Reference |
| IRF | 0.98 (0.93, 1.04) | 1.05 (0.98, 1.12) | 0.83 (0.78, 0.88) | 0.93 (0.87, 0.99) |
| HHC | 0.98 (0.93, 1.04) | 1.11 (1.04, 1.18) | 0.72 (0.68, 0.76) | 0.84 (0.79, 0.90) |
| **Axial Fracture** | | | | |
| SNF | Reference | Reference | Reference | Reference |
| IRF | 0.99 (0.94, 1.04) | 1.09 (1.02, 1.16) | 0.83 (0.78, 0.87) | 0.96 (0.90, 1.02) |
| HHC | 0.98 (0.93, 1.03) | 1.13 (1.06, 1.20) | 0.71 (0.67, 0.75) | 0.87 (0.81, 0.92) |
| **Upper Extremity Fracture** | | | | |
| SNF | Reference | Reference | Reference | Reference |
| IRF | 1.07 (0.98, 1.16) | 1.12 (1.01, 1.24) | 0.89 (0.81, 0.97) | 1.01 (0.91, 1.12) |
| HHC | 1.03 (0.95, 1.13) | 1.11 (1.00, 1.23) | 0.74 (0.68, 0.81) | 0.85 (0.77, 0.94) |
| **Intracranial Bleeding** | | | | |
| SNF | Reference | Reference | Reference | Reference |
| IRF | 1.06 (0.97, 1.18) | 1.19 (1.05, 1.34) | 0.89 (0.81, 0.99) | 1.06 (0.95, 1.20) |
| HHC | 0.97 (0.88, 1.08) | 1.08 (0.95, 1.20) | 0.71 (0.64, 0.79) | 0.84 (0.75, 0.95) |
| **Abbreviations**: CL, confidence limits; FRIs, fall-related injuries; HHC, Home Health Care; IPW, inverse probability of treatment weighting; IRF, Inpatient Rehabilitation Facilities; SNF, Skilled Nursing Facilities. | | | | |

**Table S6. Associations Between Post-Acute Care Setting Following Hip Fracture and Outcomes, Accounting for the Competing Risk of Death.**

| **Setting** | **Adjusted Hazard Ratio (95% CLs)^a^** |
| --- | --- |
| **FRIs** | |
| SNF | Reference |
| IRF | 1.01 (0.98, 1.04) |
| HHC | 0.88 (0.85, 0.90) |
| **Hospital Readmission** | |
| SNF | Reference |
| IRF | 1.00 (0.99, 1.02) |
| HHC | 0.84 (0.83, 0.85) |
| **Abbreviations:** CL, confidence limits; FRIs, fall-related injuries; HHC, Home Health Care; IRF, Inpatient Rehabilitation Facilities; SNF, Skilled Nursing Facilities.  ^a^Adjusted for all covariates in Tables 1 and 2. | |

**Table S7. E-values for quantitative bias sensitivity analyses.**

| **Setting** | **Crude** | | **IPW** | | **Crude** | | **IPW** | |
| --- | --- | --- | --- | --- | --- | --- | --- | --- |
|  | **Hazard Ratio (95% CLs)** | **E-value, Point Estimate (CL)^a^** | **Hazard Ratio (95% CLs)** | **E-value, Point Estimate (CL)^a^** | **Risk Ratio (95% CLs)** | **E-value, Point Estimate (CL)^a^** | **Risk Ratio (95% CLs)** | **E-value, Point Estimate (CL)^a^** |
| **Note: SNF is the comparator group** | | | | | | | | |
| **FRIs** | | | | | | | | |
| IRF | 0.88 (0.86, 0.90) | 1.53 (1.46) | 1.05 (1.02, 1.08) | 1.28 (1.16) | 1.04 (1.01, 1.06) | 1.24 (1.11) | 1.16 (1.13, 1.19) | 1.59 (1.51) |
| HHC | 0.76 (0.74, 0.78) | 1.96 (1.88) | 0.94 (0.92, 0.97) | 1.32 (1.21) | 1.02 (0.99, 1.04) | 1.16 (1.00**) | 1.19 (1.16, 1.22) | 1.67 (1.59) |
| **Hospital Readmission** | | | | | | | | |
| IRF | 0.89 (0.88, 0.90) | 1.39 (1.36) | 1.00 (0.99, 1.02) | 1.00 (1.00**) | 1.02 (1.01, 1.03) | 1.16 (1.11) | 1.08 (1.07, 1.09) | 1.37 (1.34) |
| HHC | 0.70 (0.70, 0.71) | 1.88 (1.85) | 0.82 (0.81, 0.83) | 1.56 (1.53) | 0.92 (0.91, 0.93) | 1.39 (1.36) | 1.00 (0.99, 1.01) | 1.00 (1.00**) |
| **Death** | | | | | | | | |
| IRF | 0.30 (0.29, 0.31) | 6.12 (5.91) | 0.40 (0.38, 0.42) | 4.44 (4.19) | 0.34 (0.33, 0.35) | 5.33 (5.16) | 0.44 (0.42, 0.45) | 3.97 (3.87) |
| HHC | 0.36 (0.35, 0.37) | 5.00 (4.85) | 0.50 (0.49, 0.52) | 3.41 (3.26) | 0.46 (0.45, 0.48) | 3.77 (3.59) | 0.61 (0.59, 0.64) | 2.66 (2.50) |
| **Hip Fracture** | | | | | | | | |
| IRF | 0.93 (0.90, 0.97) | 1.36 (1.21) | 1.16 (1.11, 1.21) | 1.59 (1.46) | 1.10 (1.06, 1.13) | 1.43 (1.31) | 1.27 (1.22, 1.32) | 1.86 (1.74) |
| HHC | 0.83 (0.80, 0.86) | 1.70 (1.60) | 1.09 (1.04, 1.13) | 1.40 (1.24) | 1.10 (1.06, 1.14) | 1.43 (1.31) | 1.34 (1.29, 1.40) | 2.01 (1.90) |
| **Lower Extremity Fracture** | | | | | | | | |
| IRF | 0.83 (0.78, 0.88) | 1.70 (1.53) | 0.93 (0.87, 0.99) | 1.36 (1.11) | 0.98 (0.93, 1.04) | 1.16 (1.00**) | 1.05 (0.98, 1.12) | 1.28 (1.00**) |
| HHC | 0.72 (0.68, 0.76) | 2.12 (1.96) | 0.84 (0.79, 0.90) | 1.67 (1.46) | 0.98 (0.93, 1.04) | 1.16 (1.00**) | 1.11 (1.04, 1.18) | 1.46 (1.24) |
| **Axial Fracture** | | | | | | | | |
| IRF | 0.83 (0.78, 0.87) | 1.70 (1.56) | 0.96 (0.90, 1.02) | 1.25 (1.00**) | 0.99 (0.94, 1.04) | 1.11 (1.00**) | 1.09 (1.02, 1.16) | 1.40 (1.16) |
| HHC | 0.71 (0.67, 0.75) | 2.17 (2.00) | 0.87 (0.81, 0.92) | 1.56 (1.39) | 0.98 (0.93, 1.03) | 1.16 (1.00**) | 1.13 (1.06, 1.20) | 1.51 (1.31) |
| **Upper Extremity Fracture** | | | | | | | | |
| IRF | 0.89 (0.81, 0.97) | 1.50 (1.21) | 1.01 (0.91, 1.12) | 1.11 (1.00**) | 1.07 (0.98, 1.16) | 1.34 (1.00**) | 1.12 (1.01, 1.24) | 1.49 (1.11) |
| HHC | 0.74 (0.68, 0.81) | 2.04 (1.77) | 0.85 (0.77, 0.94) | 1.63 (1.32) | 1.03 (0.95, 1.13) | 1.21 (1.00**) | 1.11 (1.00, 1.23) | 1.46 (1.00**) |
| **Intracranial Bleeding** | | | | | | | | |
| IRF | 0.89 (0.81, 0.99) | 1.50 (1.11) | 1.06 (0.95, 1.20) | 1.31 (1.00**) | 1.06 (0.97, 1.18) | 1.31 (1.00**) | 1.19 (1.05, 1.34) | 1.67 (1.28) |
| HHC | 0.71 (0.64, 0.79) | 2.17 (1.85) | 0.84 (0.75, 0.95) | 1.67 (1.29) | 0.97 (0.88, 1.08) | 1.21 (1.00**) | 1.08 (0.95, 1.20) | 1.37 (1.00**) |
| **Abbreviations**: CL, confidence limits; FRIs, fall-related injuries; HHC, Home Health Care; IPW, inverse probability of treatment weighted; IRF, Inpatient Rehabilitation Facilities; SNF, Skilled Nursing Facilities.  ^a^It is only meaningful to calculate the E-value for the confidence limit closest to the null (HR=1 or RR=1).  **Value is 1 because HR or RR 95% CI estimate includes 1. | | | | | | | | |
